# 2-{[{2-Hy­droxy-3-[2-methyl-5-(propan-2-yl)phen­oxy]prop­yl}(pyridin-2-ylmeth­yl)amino]­meth­yl}phenol

**DOI:** 10.1107/S1600536811020952

**Published:** 2011-06-11

**Authors:** Rakesh S. Sancheti, Amol G. Dikundwar, Ratnamala S. Bendre

**Affiliations:** a School of Chemical Sciences, North Maharashtra University, Jalgaon 425 001, India; b Solid State and Structural Chemistry Unit, Indian Institute of Science, Bangalore 560 012, Karnataka, India

## Abstract

In the title racemic compound, C_26_H_32_N_2_O_3_, an intra­molecular O—H⋯N hydrogen bond is formed between the phenolic OH group and the tertiary amine N atom. Another O—H⋯N hydrogen bond that is formed between the OH group and the pyridine N atom links the mol­ecules into a polymeric chain extending along the *a* axis. The structure is further stabilized by intramolecular and intermolecular C—H⋯O interactions.

## Related literature

For the synthesis of the title compound, see: Rossi *et al.* (2005 ▶). For related structures, see: Butcher *et al.* (2005 ▶, 2007 ▶). For the activities of related metal complexes, see: Ruiz *et al.* (2010 ▶); Yajima *et al.* (2002 ▶); Sarkar *et al.* (2006 ▶); Neves *et al.* (1999 ▶).
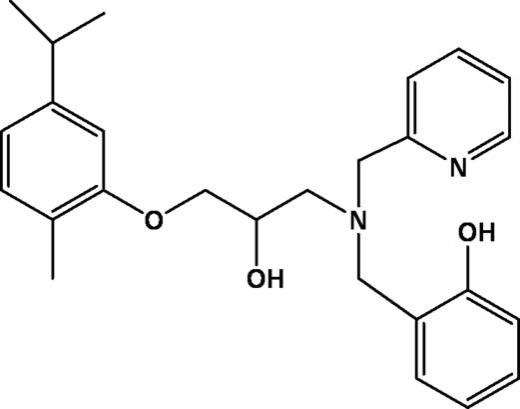


## Experimental

### 

#### Crystal data

C_26_H_32_N_2_O_3_
*M* *_r_* = 420.54
Triclinic, 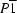

*a* = 8.0940 (6) Å
*b* = 11.3611 (7) Å
*c* = 13.7625 (10) Å
α = 79.944 (6)°
β = 82.915 (6)°
γ = 71.745 (6)°
*V* = 1180.21 (15) Å^3^
*Z* = 2
Mo *K*α radiation
μ = 0.08 mm^−1^
*T* = 120 K
0.30 × 0.30 × 0.20 mm

#### Data collection

Oxford Xcalibur Eos (Nova) CCD detector diffractometer
Absorption correction: multi-scan (*CrysAlis PRO*; Oxford Diffraction, 2009 ▶) *T*_min_ = 0.977, *T*_max_ = 0.985
23864 measured reflections
4083 independent reflections
3232 reflections with *I* > 2σ(*I*)
*R*_int_ = 0.063

#### Refinement

*R*[*F* ^2^ > 2σ(*F* ^2^)] = 0.056
*wR*(*F* ^2^) = 0.163
*S* = 1.06
4083 reflections
285 parameters
H-atom parameters constrained
Δρ_max_ = 0.99 e Å^−3^
Δρ_min_ = −0.27 e Å^−3^

### 

Data collection: *CrysAlis PRO* (Oxford Diffraction, 2009 ▶); cell refinement: *CrysAlis PRO*; data reduction: *CrysAlis PRO*; program(s) used to solve structure: *SHELXS97* (Sheldrick, 2008 ▶); program(s) used to refine structure: *SHELXL97* (Sheldrick, 2008 ▶); molecular graphics: *ORTEP-3* (Farrugia, 1997 ▶) and *CAMERON* (Watkin *et al.*, 1993 ▶); software used to prepare material for publication: *WinGX* (Farrugia, 1999 ▶).

## Supplementary Material

Crystal structure: contains datablock(s) global, I. DOI: 10.1107/S1600536811020952/gk2373sup1.cif

Structure factors: contains datablock(s) I. DOI: 10.1107/S1600536811020952/gk2373Isup2.hkl

Supplementary material file. DOI: 10.1107/S1600536811020952/gk2373Isup3.cml

Additional supplementary materials:  crystallographic information; 3D view; checkCIF report

## Figures and Tables

**Table 1:** Hydrogen-bond geometry (Å, °)

*D*—H⋯*A*	*D*—H	H⋯*A*	*D*⋯*A*	*D*—H⋯*A*
O2—H2O⋯N2^i^	0.82	2.09	2.897 (2)	166
O3—H3⋯N1	0.82	1.99	2.721 (2)	147
C14—H14*A*⋯O2	0.97	2.51	3.199 (3)	128
C23—H23⋯O3^ii^	0.93	2.47	3.114 (3)	127

Symmetry codes: (i) 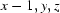; (ii) 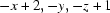.

**Fig. 1.**
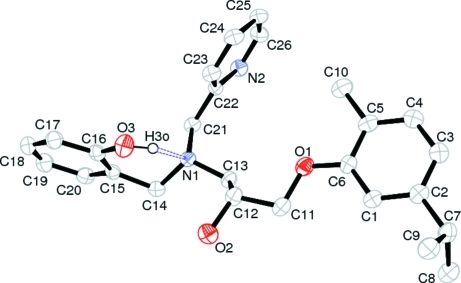
Molecular structure of the title compound showing intramolecular O—H···N hydrogen bond. Displacement ellipsoids are drawn at the 50% probability level. The H atom involved in intramolecular hydrogen bond is shown as a sphere of arbitrary radius.

**Fig. 2.**
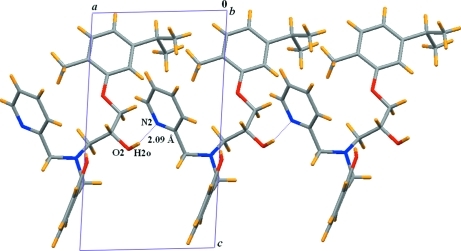
Packing of the title compound viewed down the b axis. The dotted lines indicate intermolecular O—H···N interactions.

## supplementary crystallographic information

### Comment

The chemistry of asymmetric polydentate ligands evokes interest, mainly towards the synthesis of biologically active coordination compounds. DNA metallointercalators have received considerable attention over the past few years because of their possible uses as new therapeutic agents and also for their interesting photochemical properties (Ruiz *et al.*, 2010; Yajima *et al.*, 2002; Sarkar *et al.*, 2006). There are several reports on copper complexes of the asymmetrical ligands exhibiting important biological activities such as genomic and plasmid DNA cleavage and cytotoxic activity (Neves *et al.*, 1999; Rossi *et al.*, 2005).

The title compound was synthesized for preparation of metal complexes which would act as chemical nucleases. The ligand coordinates with a metal ion through its N_2_O_2_ donor set along with an additional halide ligand to form a complex with distorted trigonal bipyramidal geometry. The molecular conformation of the ligand is nonplanar with O—H···N and C—H···O intramolecular hydrogen bonds, both forming the six-membered rings (Fig. 1, Table 1). Packing of the molecules is mainly guided by the intermolecular O—H···N hydrogen bonds connecting the 1-(5-isopropyl-2-methylphenoxy)propan-2-ol fragment of one molecule to the pyridine fragment of the other.

### Experimental

The title compound was synthesized by the reaction of 2-[(5-isopropyl-2-methylphenoxy)methyl]oxirane (5.8 mmol, 1.20 g) with *N*-(2-hydroxybenzyl)-*N*-(2-pyridylmethyl)amine (5.8 mmol, 1.23 g) in methanol under reflux condition at 70°C for 8 h. The reaction mixture was cooled, filtered and the precipitated product was washed with cold methanol in order to remove the impurities (yield 66%, m.p. 407K). Crystals suitable for X-ray diffraction were obtained by slow evaporation of the saturated solution in acetonitrile at room temperature.

### Refinement

All H atoms were positioned geometrically (C—H = 0.93-0.97 Å, O—H = 0.82 Å) and refined using a riding model with *U*_iso_(H)= 1.2*U*_eq_(C,O).

The high residual peak of 0.99 e Å^-3^ observed in a difference map was located at a distance of 1.05 Å from C12 and it may represent O atom of the OH group of the opposite enantiomer located at the same site in crystal. No reasonable model of the disorder could be obtained as the occupancy of the minor enantiomer should be only a few percent, with a significant overlap of the atomic positions.

### Crystal data

**Table:**

C_26_H_32_N_2_O_3_	*Z* = 2
*M**_r_* = 420.54	*F*(000) = 452
Triclinic, *P*1	*D*_x_ = 1.183 Mg m^−^^3^
Hall symbol: -P 1	Mo *K*α radiation, λ = 0.71073 Å
*a* = 8.0940 (6) Å	Cell parameters from 23864 reflections
*b* = 11.3611 (7) Å	θ = 2.6–25.0°
*c* = 13.7625 (10) Å	µ = 0.08 mm^−^^1^
α = 79.944 (6)°	*T* = 120 K
β = 82.915 (6)°	Block, colourless
γ = 71.745 (6)°	0.30 × 0.30 × 0.20 mm
*V* = 1180.21 (15) Å^3^	

### Data collection

**Table:**

Oxford Xcalibur Eos (Nova) CCD detector diffractometer	4083 independent reflections
Radiation source: Enhance (Mo) X-ray Source	3232 reflections with *I* > 2σ(*I*)
graphite	*R*_int_ = 0.063
ω scans	θ_max_ = 25.0°, θ_min_ = 2.6°
Absorption correction: multi-scan (*CrysAlis PRO*; Oxford Diffraction, 2009)	*h* = −9→9
*T*_min_ = 0.977, *T*_max_ = 0.985	*k* = −13→13
23864 measured reflections	*l* = −16→16

### Refinement

**Table:**

Refinement on *F*^2^	Primary atom site location: structure-invariant direct methods
Least-squares matrix: full	Secondary atom site location: difference Fourier map
*R*[*F*^2^ > 2σ(*F*^2^)] = 0.056	Hydrogen site location: inferred from neighbouring sites
*wR*(*F*^2^) = 0.163	H-atom parameters constrained
*S* = 1.06	*w* = 1/[σ^2^(*F*_o_^2^) + (0.0984*P*)^2^ + 0.1634*P*] where *P* = (*F*_o_^2^ + 2*F*_c_^2^)/3
4083 reflections	(Δ/σ)_max_ < 0.001
285 parameters	Δρ_max_ = 0.99 e Å^−^^3^
0 restraints	Δρ_min_ = −0.27 e Å^−^^3^

### Special details

**Table:**

Experimental. 1H NMR (p.p.m., CDCl_3_): 1.20 (d, 6H, 2-CH_3_), 2.08 (s, 3H, –CH_3_), 2.84 (d, 2H, –CH_2_), 3.10 (m, 1H, –CH), 3.90 (m, 4H 2-CH_2_), 4.10(d, 2H, –CH_2_), 4.25 (m, 1H, –CH), 4.58 (bs, 1H, Ar—OH), 6.7 to 7.7 Ar—H. (Found: C 74.54, H 7.37, N 6.67%; Calcd. for C_26_H_31_O_3_N_2_: C 74.44, H 7.44, N 7.05%) IR (cm-l): ? (C═C) 1586, ? (C—O—C) 1151. MS (m/z): 421[M]+
Geometry. All esds (except the esd in the dihedral angle between two l.s. planes) are estimated using the full covariance matrix. The cell esds are taken into account individually in the estimation of esds in distances, angles and torsion angles; correlations between esds in cell parameters are only used when they are defined by crystal symmetry. An approximate (isotropic) treatment of cell esds is used for estimating esds involving l.s. planes.
Refinement. Refinement of *F*^2^ against ALL reflections. The weighted *R*-factor *wR* and goodness of fit *S* are based on *F*^2^, conventional *R*-factors *R* are based on *F*, with *F* set to zero for negative *F*^2^. The threshold expression of *F*^2^ > σ(*F*^2^) is used only for calculating *R*-factors(gt) *etc*. and is not relevant to the choice of reflections for refinement. *R*-factors based on *F*^2^ are statistically about twice as large as those based on *F*, and *R*-factors based on ALL data will be even larger.

### Fractional atomic coordinates and isotropic or equivalent isotropic displacement parameters (Å^2^)

**Table:**

	*x*	*y*	*z*	*U*_iso_*/*U*_eq_	
C1	0.7021 (3)	0.58331 (18)	0.23411 (15)	0.0272 (5)	
H1	0.6049	0.5897	0.2789	0.033*	
C2	0.6851 (3)	0.65628 (19)	0.14101 (15)	0.0289 (5)	
C3	0.8326 (3)	0.6470 (2)	0.07647 (15)	0.0308 (5)	
H3A	0.8240	0.6956	0.0144	0.037*	
C4	0.9931 (3)	0.5658 (2)	0.10358 (16)	0.0317 (5)	
H4	1.0904	0.5613	0.0591	0.038*	
C5	1.0125 (3)	0.49111 (18)	0.19502 (16)	0.0287 (5)	
C6	0.8624 (3)	0.50136 (18)	0.26044 (15)	0.0260 (5)	
C7	0.5086 (3)	0.7432 (2)	0.11150 (16)	0.0323 (5)	
H7	0.5243	0.7825	0.0431	0.039*	
C8	0.4387 (3)	0.8471 (2)	0.17527 (18)	0.0375 (5)	
H8A	0.4249	0.8112	0.2432	0.056*	
H8B	0.3278	0.9006	0.1540	0.056*	
H8C	0.5191	0.8952	0.1689	0.056*	
C9	0.3783 (3)	0.6693 (2)	0.11357 (17)	0.0360 (5)	
H9A	0.4214	0.6093	0.0683	0.054*	
H9B	0.2678	0.7260	0.0945	0.054*	
H9C	0.3643	0.6266	0.1792	0.054*	
C10	1.1857 (3)	0.4025 (2)	0.22438 (18)	0.0381 (5)	
H10A	1.2731	0.4077	0.1712	0.057*	
H10B	1.1780	0.3185	0.2383	0.057*	
H10C	1.2168	0.4246	0.2823	0.057*	
C11	0.7519 (3)	0.43452 (19)	0.42513 (16)	0.0290 (5)	
H11A	0.6481	0.4302	0.3992	0.035*	
H11B	0.7242	0.5131	0.4511	0.035*	
C12	0.8149 (3)	0.32503 (19)	0.50560 (16)	0.0300 (5)	
H12	0.8512	0.2478	0.4757	0.036*	
C13	0.9716 (3)	0.33436 (18)	0.55172 (15)	0.0249 (4)	
H13A	1.0516	0.3589	0.4995	0.030*	
H13B	0.9321	0.3992	0.5940	0.030*	
C14	0.9973 (3)	0.20672 (18)	0.71470 (14)	0.0253 (4)	
H14A	0.8710	0.2386	0.7183	0.030*	
H14B	1.0401	0.2583	0.7484	0.030*	
C15	1.0514 (2)	0.07363 (18)	0.76661 (14)	0.0247 (4)	
C16	1.0327 (3)	−0.02368 (19)	0.72237 (15)	0.0271 (5)	
C17	1.0661 (3)	−0.1443 (2)	0.77277 (17)	0.0345 (5)	
H17	1.0498	−0.2074	0.7436	0.041*	
C18	1.1244 (3)	−0.1707 (2)	0.86749 (17)	0.0392 (6)	
H18	1.1463	−0.2515	0.9018	0.047*	
C19	1.1499 (3)	−0.0776 (2)	0.91064 (17)	0.0404 (6)	
H19	1.1928	−0.0960	0.9729	0.048*	
C20	1.1114 (3)	0.0433 (2)	0.86082 (15)	0.0326 (5)	
H20	1.1260	0.1061	0.8912	0.039*	
C21	1.2544 (2)	0.19811 (18)	0.60307 (14)	0.0239 (4)	
H21A	1.3081	0.1279	0.6517	0.029*	
H21B	1.2744	0.2722	0.6189	0.029*	
C22	1.3400 (2)	0.17410 (17)	0.50250 (14)	0.0227 (4)	
C23	1.2875 (3)	0.10394 (18)	0.44568 (15)	0.0273 (5)	
H23	1.1955	0.0716	0.4694	0.033*	
C24	1.3720 (3)	0.08254 (19)	0.35424 (15)	0.0290 (5)	
H24	1.3369	0.0368	0.3153	0.035*	
C25	1.5104 (3)	0.13057 (19)	0.32132 (16)	0.0298 (5)	
H25	1.5707	0.1177	0.2602	0.036*	
C26	1.5555 (3)	0.19783 (19)	0.38222 (15)	0.0296 (5)	
H26	1.6486	0.2295	0.3603	0.036*	
N1	1.0650 (2)	0.21620 (14)	0.61015 (12)	0.0226 (4)	
N2	1.4744 (2)	0.22078 (16)	0.47087 (13)	0.0275 (4)	
O1	0.89021 (18)	0.42567 (13)	0.34963 (11)	0.0313 (4)	
O2	0.67674 (19)	0.31938 (15)	0.57718 (11)	0.0354 (4)	
H2O	0.6056	0.2961	0.5541	0.053*	
O3	0.9810 (2)	−0.00092 (14)	0.62858 (11)	0.0341 (4)	
H3	0.9930	0.0662	0.6008	0.051*	

### Atomic displacement parameters (Å^2^)

**Table:**

	*U*^11^	*U*^22^	*U*^33^	*U*^12^	*U*^13^	*U*^23^
C1	0.0266 (11)	0.0251 (10)	0.0336 (11)	−0.0092 (9)	−0.0063 (9)	−0.0082 (9)
C2	0.0310 (11)	0.0274 (11)	0.0339 (11)	−0.0117 (9)	−0.0080 (9)	−0.0095 (9)
C3	0.0360 (12)	0.0321 (11)	0.0292 (11)	−0.0144 (10)	−0.0062 (9)	−0.0069 (9)
C4	0.0331 (12)	0.0330 (12)	0.0339 (12)	−0.0137 (9)	−0.0015 (9)	−0.0118 (9)
C5	0.0284 (11)	0.0240 (11)	0.0388 (12)	−0.0095 (9)	−0.0058 (9)	−0.0130 (9)
C6	0.0299 (11)	0.0211 (10)	0.0311 (11)	−0.0110 (8)	−0.0077 (9)	−0.0046 (8)
C7	0.0294 (12)	0.0343 (12)	0.0348 (12)	−0.0112 (9)	−0.0108 (9)	0.0002 (9)
C8	0.0324 (12)	0.0311 (12)	0.0490 (14)	−0.0083 (10)	−0.0068 (10)	−0.0054 (10)
C9	0.0305 (12)	0.0404 (13)	0.0405 (13)	−0.0115 (10)	−0.0118 (10)	−0.0067 (10)
C10	0.0310 (12)	0.0325 (12)	0.0520 (14)	−0.0095 (10)	−0.0037 (10)	−0.0089 (10)
C11	0.0221 (10)	0.0265 (11)	0.0411 (12)	−0.0101 (8)	−0.0090 (9)	−0.0017 (9)
C12	0.0243 (11)	0.0282 (11)	0.0400 (12)	−0.0124 (9)	−0.0072 (9)	0.0004 (9)
C13	0.0250 (10)	0.0218 (10)	0.0299 (11)	−0.0093 (8)	−0.0039 (8)	−0.0037 (8)
C14	0.0224 (10)	0.0273 (11)	0.0295 (11)	−0.0098 (8)	−0.0028 (8)	−0.0080 (8)
C15	0.0205 (10)	0.0268 (11)	0.0284 (11)	−0.0096 (8)	0.0007 (8)	−0.0051 (8)
C16	0.0210 (10)	0.0309 (11)	0.0324 (11)	−0.0130 (8)	0.0009 (8)	−0.0049 (9)
C17	0.0313 (12)	0.0284 (12)	0.0455 (14)	−0.0137 (9)	0.0033 (10)	−0.0056 (10)
C18	0.0390 (13)	0.0298 (12)	0.0408 (13)	−0.0083 (10)	0.0062 (10)	0.0056 (10)
C19	0.0426 (14)	0.0411 (14)	0.0304 (12)	−0.0066 (11)	−0.0015 (10)	0.0017 (10)
C20	0.0313 (12)	0.0357 (12)	0.0307 (11)	−0.0089 (9)	−0.0001 (9)	−0.0079 (9)
C21	0.0192 (10)	0.0240 (10)	0.0324 (11)	−0.0092 (8)	−0.0057 (8)	−0.0064 (8)
C22	0.0180 (9)	0.0196 (10)	0.0316 (11)	−0.0063 (8)	−0.0064 (8)	−0.0018 (8)
C23	0.0221 (10)	0.0275 (11)	0.0375 (12)	−0.0123 (8)	−0.0034 (9)	−0.0083 (9)
C24	0.0245 (10)	0.0284 (11)	0.0370 (12)	−0.0064 (9)	−0.0058 (9)	−0.0131 (9)
C25	0.0230 (10)	0.0302 (11)	0.0348 (12)	−0.0049 (9)	−0.0009 (9)	−0.0076 (9)
C26	0.0233 (10)	0.0336 (12)	0.0362 (12)	−0.0141 (9)	0.0013 (9)	−0.0080 (9)
N1	0.0183 (8)	0.0230 (8)	0.0288 (9)	−0.0082 (7)	−0.0039 (7)	−0.0042 (7)
N2	0.0230 (9)	0.0280 (9)	0.0362 (10)	−0.0127 (7)	−0.0032 (7)	−0.0073 (7)
O1	0.0266 (8)	0.0294 (8)	0.0365 (8)	−0.0063 (6)	−0.0073 (6)	−0.0015 (6)
O2	0.0309 (9)	0.0410 (9)	0.0389 (9)	−0.0168 (7)	−0.0047 (7)	−0.0051 (7)
O3	0.0400 (9)	0.0315 (8)	0.0405 (9)	−0.0206 (7)	−0.0123 (7)	−0.0054 (7)

### Geometric parameters (Å, °)

**Table:**

C1—C6	1.386 (3)	C13—H13B	0.9700
C1—C2	1.396 (3)	C14—N1	1.473 (3)
C1—H1	0.9300	C14—C15	1.508 (3)
C2—C3	1.384 (3)	C14—H14A	0.9700
C2—C7	1.516 (3)	C14—H14B	0.9700
C3—C4	1.388 (3)	C15—C20	1.389 (3)
C3—H3A	0.9300	C15—C16	1.407 (3)
C4—C5	1.386 (3)	C16—O3	1.362 (2)
C4—H4	0.9300	C16—C17	1.384 (3)
C5—C6	1.406 (3)	C17—C18	1.392 (3)
C5—C10	1.502 (3)	C17—H17	0.9300
C6—O1	1.369 (2)	C18—C19	1.378 (3)
C7—C8	1.518 (3)	C18—H18	0.9300
C7—C9	1.535 (3)	C19—C20	1.382 (3)
C7—H7	0.9800	C19—H19	0.9300
C8—H8A	0.9600	C20—H20	0.9300
C8—H8B	0.9600	C21—N1	1.474 (2)
C8—H8C	0.9600	C21—C22	1.499 (3)
C9—H9A	0.9600	C21—H21A	0.9700
C9—H9B	0.9600	C21—H21B	0.9700
C9—H9C	0.9600	C22—N2	1.348 (3)
C10—H10A	0.9600	C22—C23	1.393 (3)
C10—H10B	0.9600	C23—C24	1.379 (3)
C10—H10C	0.9600	C23—H23	0.9300
C11—O1	1.422 (3)	C24—C25	1.389 (3)
C11—C12	1.515 (3)	C24—H24	0.9300
C11—H11A	0.9700	C25—C26	1.376 (3)
C11—H11B	0.9700	C25—H25	0.9300
C12—O2	1.406 (3)	C26—N2	1.339 (3)
C12—C13	1.526 (3)	C26—H26	0.9300
C12—H12	0.9800	O2—H2O	0.8200
C13—N1	1.469 (2)	O3—H3	0.8200
C13—H13A	0.9700		
			
C6—C1—C2	120.7 (2)	C12—C13—H13A	109.0
C6—C1—H1	119.7	N1—C13—H13B	109.0
C2—C1—H1	119.7	C12—C13—H13B	109.0
C3—C2—C1	118.5 (2)	H13A—C13—H13B	107.8
C3—C2—C7	120.99 (19)	N1—C14—C15	111.99 (16)
C1—C2—C7	120.52 (19)	N1—C14—H14A	109.2
C2—C3—C4	120.6 (2)	C15—C14—H14A	109.2
C2—C3—H3A	119.7	N1—C14—H14B	109.2
C4—C3—H3A	119.7	C15—C14—H14B	109.2
C5—C4—C3	121.9 (2)	H14A—C14—H14B	107.9
C5—C4—H4	119.1	C20—C15—C16	117.71 (18)
C3—C4—H4	119.1	C20—C15—C14	121.98 (18)
C4—C5—C6	117.28 (19)	C16—C15—C14	120.21 (17)
C4—C5—C10	122.1 (2)	O3—C16—C17	118.51 (19)
C6—C5—C10	120.66 (19)	O3—C16—C15	120.51 (18)
O1—C6—C1	124.48 (19)	C17—C16—C15	120.97 (19)
O1—C6—C5	114.48 (18)	C16—C17—C18	119.5 (2)
C1—C6—C5	121.04 (19)	C16—C17—H17	120.2
C2—C7—C8	111.83 (17)	C18—C17—H17	120.2
C2—C7—C9	110.83 (17)	C19—C18—C17	120.3 (2)
C8—C7—C9	111.36 (19)	C19—C18—H18	119.8
C2—C7—H7	107.5	C17—C18—H18	119.8
C8—C7—H7	107.5	C18—C19—C20	119.7 (2)
C9—C7—H7	107.5	C18—C19—H19	120.2
C7—C8—H8A	109.5	C20—C19—H19	120.2
C7—C8—H8B	109.5	C19—C20—C15	121.7 (2)
H8A—C8—H8B	109.5	C19—C20—H20	119.1
C7—C8—H8C	109.5	C15—C20—H20	119.1
H8A—C8—H8C	109.5	N1—C21—C22	112.97 (15)
H8B—C8—H8C	109.5	N1—C21—H21A	109.0
C7—C9—H9A	109.5	C22—C21—H21A	109.0
C7—C9—H9B	109.5	N1—C21—H21B	109.0
H9A—C9—H9B	109.5	C22—C21—H21B	109.0
C7—C9—H9C	109.5	H21A—C21—H21B	107.8
H9A—C9—H9C	109.5	N2—C22—C23	121.50 (19)
H9B—C9—H9C	109.5	N2—C22—C21	116.56 (16)
C5—C10—H10A	109.5	C23—C22—C21	121.91 (17)
C5—C10—H10B	109.5	C24—C23—C22	119.90 (19)
H10A—C10—H10B	109.5	C24—C23—H23	120.1
C5—C10—H10C	109.5	C22—C23—H23	120.1
H10A—C10—H10C	109.5	C23—C24—C25	118.79 (19)
H10B—C10—H10C	109.5	C23—C24—H24	120.6
O1—C11—C12	106.52 (16)	C25—C24—H24	120.6
O1—C11—H11A	110.4	C26—C25—C24	117.8 (2)
C12—C11—H11A	110.4	C26—C25—H25	121.1
O1—C11—H11B	110.4	C24—C25—H25	121.1
C12—C11—H11B	110.4	N2—C26—C25	124.49 (19)
H11A—C11—H11B	108.6	N2—C26—H26	117.8
O2—C12—C11	109.42 (16)	C25—C26—H26	117.8
O2—C12—C13	111.46 (17)	C13—N1—C14	112.26 (15)
C11—C12—C13	111.23 (16)	C13—N1—C21	111.67 (15)
O2—C12—H12	108.2	C14—N1—C21	110.08 (15)
C11—C12—H12	108.2	C26—N2—C22	117.52 (17)
C13—C12—H12	108.2	C6—O1—C11	119.89 (15)
N1—C13—C12	112.82 (15)	C12—O2—H2O	109.5
N1—C13—H13A	109.0	C16—O3—H3	109.5
			
C6—C1—C2—C3	−1.4 (3)	O3—C16—C17—C18	177.95 (19)
C6—C1—C2—C7	178.34 (17)	C15—C16—C17—C18	−2.0 (3)
C1—C2—C3—C4	0.7 (3)	C16—C17—C18—C19	−0.5 (3)
C7—C2—C3—C4	−179.02 (18)	C17—C18—C19—C20	2.3 (3)
C2—C3—C4—C5	0.2 (3)	C18—C19—C20—C15	−1.7 (3)
C3—C4—C5—C6	−0.5 (3)	C16—C15—C20—C19	−0.7 (3)
C3—C4—C5—C10	179.60 (18)	C14—C15—C20—C19	175.7 (2)
C2—C1—C6—O1	−179.35 (17)	N1—C21—C22—N2	145.52 (17)
C2—C1—C6—C5	1.2 (3)	N1—C21—C22—C23	−36.4 (2)
C4—C5—C6—O1	−179.73 (17)	N2—C22—C23—C24	−1.0 (3)
C10—C5—C6—O1	0.2 (3)	C21—C22—C23—C24	−178.94 (18)
C4—C5—C6—C1	−0.2 (3)	C22—C23—C24—C25	0.9 (3)
C10—C5—C6—C1	179.71 (18)	C23—C24—C25—C26	−0.2 (3)
C3—C2—C7—C8	−115.7 (2)	C24—C25—C26—N2	−0.4 (3)
C1—C2—C7—C8	64.5 (3)	C12—C13—N1—C14	−90.37 (19)
C3—C2—C7—C9	119.4 (2)	C12—C13—N1—C21	145.45 (17)
C1—C2—C7—C9	−60.4 (3)	C15—C14—N1—C13	162.17 (15)
O1—C11—C12—O2	−172.92 (15)	C15—C14—N1—C21	−72.78 (19)
O1—C11—C12—C13	63.5 (2)	C22—C21—N1—C13	−69.2 (2)
O2—C12—C13—N1	72.9 (2)	C22—C21—N1—C14	165.45 (15)
C11—C12—C13—N1	−164.73 (17)	C25—C26—N2—C22	0.3 (3)
N1—C14—C15—C20	136.15 (19)	C23—C22—N2—C26	0.4 (3)
N1—C14—C15—C16	−47.5 (2)	C21—C22—N2—C26	178.44 (17)
C20—C15—C16—O3	−177.38 (18)	C1—C6—O1—C11	−4.5 (3)
C14—C15—C16—O3	6.1 (3)	C5—C6—O1—C11	175.02 (16)
C20—C15—C16—C17	2.6 (3)	C12—C11—O1—C6	170.36 (15)
C14—C15—C16—C17	−173.90 (18)		

### Hydrogen-bond geometry (Å, °)

**Table:**

*D*—H···*A*	*D*—H	H···*A*	*D*···*A*	*D*—H···*A*
O2—H2O···N2^i^	0.82	2.09	2.897 (2)	166
O3—H3···N1	0.82	1.99	2.721 (2)	147
C14—H14A···O2	0.97	2.51	3.199 (3)	128
C23—H23···O3^ii^	0.93	2.47	3.114 (3)	127

Symmetry codes: (i) *x*−1, *y*, *z*; (ii) −*x*+2, −*y*, −*z*+1.
